# JAK Inhibitors in Colchicine Resistant FMF Patients: A Case-Based Review

**DOI:** 10.31138/mjr.281125.mrk

**Published:** 2026-09-01

**Authors:** Farhad Salehzadeh, Rasol Molatefi, Faeze Babazadeh Khoei

**Affiliations:** Paediatric Department, Bouali Children`s Hospital, Ardabil University of Medical Sciences (ARUMS), Ardabil, Iran

**Keywords:** JAK Inhibitor, colchicine resistant FMF, Familial Mediterranean Fever

## Abstract

**Background:**

Familial Mediterranean Fever (FMF) is an autoinflammatory disorder typically controlled with colchicine; however, 5–10% of patients exhibit colchicine resistance or intolerance. Although biologic therapies targeting IL-1 may be effective, their cost and limited accessibility create a need for alternative oral treatments. Janus kinase (JAK) inhibitors have emerged as potential modulators of multiple inflammatory pathways implicated in FMF pathogenesis.

**Methods:**

We conducted a retrospective case series of three adults with colchicine-resistant FMF who had previously demonstrated partial but inadequate responses to colchicine and dapsone combination therapy. All patients-initiated treatment with an oral JAK inhibitor at 5 mg twice daily. None of the patients had received IL-1 or IL-6 inhibitors prior to JAK inhibitor therapy. Clinical outcomes, including attack frequency, remission status, and adverse events, were documented over 9–12 months of follow-up.

**Results:**

All three patients achieved complete remission, defined as the absence of any FMF attacks throughout the follow-up period. No infectious, hematologic, thrombotic, or hepatic adverse events were observed.

**Conclusion:**

JAK inhibitor therapy was associated with rapid, complete, and sustained remission in adults with colchicine-resistant FMF. These findings support the growing evidence that JAK inhibition may offer an effective oral alternative to biologics in refractory FMF.

## INTRODUCTION

Familial Mediterranean Fever (FMF) is a hereditary autoinflammatory disorder characterised by recurrent episodes of fever, peritonitis, pleuritis, and arthritis, resulting from mutations in the Mediterranean fever Gene (MEFV) gene that lead to excessive activation of the innate immune system.^1^ Colchicine remains the cornerstone of FMF treatment, effectively preventing attacks and amyloidosis in most patients; however, approximately 5–10% exhibit colchicine resistance or intolerance, posing a significant therapeutic challenge.^2^ In colchicine-resistant FMF, biologic therapies targeting interleukin-1 (IL-1) have demonstrated efficacy, yet issues related to high cost, limited accessibility, and incomplete response restrict their widespread use.^3^ In this context, the study by Salehzadeh and colleagues showed that the addition of dapsone to colchicine therapy improved clinical outcomes in colchicine-resistant FMF patients.^4^

Recent advances in understanding autoinflammatory pathways have highlighted the central role of the Janus kinase–signal transducer and activator of transcription (JAK–STAT) signalling cascade in mediating inflammatory cytokine activity, including IL-6, interferons, and granulocyte-macrophage colony-stimulating factor GM-CSF.^5^ This has led to growing interest in JAK inhibitors (JAKi) small molecules that block one or more JAK family members (JAK1, JAK2, JAK3, TYK2)—as potential agents for diseases driven by dysregulated cytokine signalling.^6^

## MATERIALS AND METHODS

This study was a retrospective case series evaluating the clinical outcomes of three patients with colchicine-resistant Familial Mediterranean Fever (FMF) who were subsequently treated with an oral Janus kinase (JAK) inhibitor at a fixed dose of 5 mg twice daily. Patients were recruited from a single rheumatology clinic (www.fmfiran.ir) and were diagnosed with FMF based on clinical criteria consistent with Tel-Hashomer definitions, supported by MEFV gene mutation analysis.

All patients included met the following criteria:

1. Recurrent FMF attacks despite maximum tolerated colchicine therapy,
2. Partial but inadequate clinical response to combined colchicine + dapsone,
3. No prior exposure to biologic agents such as IL-1 or IL-6 inhibitors.

Colchicine resistance was defined as ≥1 FMF attack per month despite maximally tolerating colchicine dose for at least 6 months. Partial response referred to reduced attack frequency but persistence of symptomatic episodes while receiving colchicine plus dapsone. Complete remission was defined as the absence of any FMF attacks after initiation of the JAK inhibitor. Before initiating JAK inhibitor therapy, all patients had received:

- Colchicine at individualised maximal tolerated doses (1.5–2.5 mg/day),
- Dapsone 100 mg/day as adjunct therapy.

Due to continued attacks, each patient was transitioned to a JAK inhibitor at 5 mg PO BID.

IL-1 inhibitors are not routinely available in our country, and its limited usage is not supported by insurance companies, so no biologic therapy (IL-1 or IL-6 blockers) was used at any point.

Clinical response was evaluated by:

- Attack frequency (number of febrile/peritonitis/serositis episodes per month),
- Transition from partial response to complete remission,
- Duration of follow-up after JAK inhibitor initiation (9–12 months).

Patients were monitored during routine clinic visits.

All three individuals achieved complete remission, defined as no attacks during the entire follow-up period. At each follow-up visit, patients were assessed for potential adverse events, including infections, cytopenias, thromboembolic events, and hepatic abnormalities. Across all three patients, no adverse effects attributable to JAK inhibitor therapy were observed.

## ETHICAL APPROVAL

The study protocol was approved by the Ethical Committee of Ardabil University of Medical Sciences, Ardabil, Iran (Code: IR.ARUMS.REC.1399.604).

Written informed consent was obtained from all participants.

## CASE PRESENTATION

We report to three adult patients with long-standing colchicine-resistant FMF, all of whom continued to experience recurrent attacks despite maximally tolerated colchicine and combination therapy with dapsone (**Table 1**).

**Table 1.** Clinical characteristics and treatment outcomes of the three colchicine-resistant FMF patients treated with a JAK inhibitor (5 mg PO BID).

	**Patient 1**	**Patient 2**	**Patient 3**
Sex / Age	Female / 52	Female / 35	Male / 50
Age at symptom onset	Second decade	Second decade	First decade
MEFV genotype	M680I / V726A (compound heterozygous)	E148Q (heterozygous)	M694V (heterozygous)
Colchicine therapy	Inadequate response	Inadequate response	No response
Dapsone therapy	Partial response (combination)	Partial response (combination)	Partial response (combination)
Biologic therapy	Not used	Not used	Not used
Reason for JAK inhibitor initiation	Persistent attacks despite colchicine + dapsone	Persistent attacks despite colchicine + dapsone	Persistent attacks despite colchicine + dapsone
JAK inhibitor dose	5 mg PO BID	5 mg PO BID	5 mg PO BID
Duration of follow-up after JAKi initiation	9 months	12 months	12 months
Clinical response to JAK inhibitor	Complete remission (no attacks)	Complete remission (no attacks)	Complete remission (no attacks)
Adverse events	None	None	None
Inflammatory Markers ESR (mm/h), CRP (mg/dL)	45/3+	40/3+	50/2+

### Case 1

A 52-year-old woman with FMF symptoms beginning in her second decade presented with recurrent febrile serositis attacks despite adherence to colchicine and subsequent combination therapy with dapsone (100 mg/day). Genetic testing revealed compound heterozygosity for M680I/V726A in the MEFV gene. Because she continued to experience attacks, although of reduced severity, she was transitioned to a Tofacitinib (5 mg PO BID). Over a 9-month follow-up period, she exhibited complete remission, with no further attacks and no treatment-related adverse effects.

### Case 2

A 35-year-old woman with disease onset in adolescence and heterozygous E148Q mutation reported persistent attacks despite colchicine at 2.5 mg/day and partial benefit from the addition of dapsone. She had not received biologics. Initiation of a Tofacitinib (5 mg PO BID) resulted in sustained remission, with complete disappearance of attacks during 12 months of follow-up and no adverse events.

### Case 3

A 50-year-old man with FMF symptoms starting in early childhood, carrying a heterozygous M694V mutation, had demonstrated poor response to colchicine and only partial improvement on combination therapy with dapsone. Due to ongoing attacks, he was started on a Tofacitinib (5 mg PO BID). During 1 year of follow-up, he remained entirely attack-free, and no adverse events were recorded.

Across all three cases, initiation of JAK inhibitor therapy led to rapid and complete cessation of attacks despite prior resistance to colchicine and suboptimal response to dapsone. No patient experienced infectious, hematologic, thrombotic, or other safety concerns during treatment.

## DISCUSSION

In this series, we describe three patients with long-standing colchicine-resistant FMF who experienced dramatic clinical responses following initiation of oral JAK inhibitor therapy.

All three patients had previously received no biologic therapies, and the dramatic, sustained responses to JAK inhibitors highlight their potential utility in colchicine–dapsone resistant FMF.

Preliminary reports and small case series indicate that JAK inhibitors, such as tofacitinib and baricitinib, may reduce attack frequency and systemic inflammation in colchicine-resistant FMF patients, offering a promising therapeutic alternative when conventional biologics are ineffective or unavailable.^7^ These findings are further supported by mechanistic evidence showing that JAK inhibition can simultaneously modulate multiple cytokine pathways, thereby overcoming cytokine redundancy observed in FMF and other autoinflammatory syndromes.^5^

Nevertheless, long-term safety concerns, including increased susceptibility to infections and viral reactivation, highlight the need for careful patient selection and larger randomised controlled trials to clarify efficacy and safety profiles.^8^ Overall, JAK inhibitors may represent a rational and effective therapeutic option for colchicine-resistant FMF, complementing earlier efforts such as the colchicine-plus-dapsone approach described by Salehzadeh et al.^4^

In this case series of three patients with long-standing colchicine-resistant Familial Mediterranean Fever (FMF), we observed that initiation of an oral JAK inhibitor at standard dosing resulted in complete cessation of attacks and striking sustained remission of disease activity.

The dramatic clinical responses in our cohort highlight an evolving therapeutic paradigm for this difficult-to-treat patient population.

Our findings reinforce the growing body of evidence that the JAK–STAT signalling pathway plays a pivotal role in autoinflammatory disorders, including FMF.^9^ Cytokine-driven inflammation is central to FMF pathogenesis, and the JAK–STAT axis mediates signalling for multiple pro-inflammatory cytokines such as IL-6 and interferon-γ.^10,11^ Banerjee et al. described the JAK– STAT pathway as a promising therapeutic target in various inflammatory and autoimmune diseases.^12^ Similarly, Xue et al. reported that JAK inhibition suppresses abnormal cytokine responses by interfering with key intracellular signalling nodes.^11^ In this context, our observation of complete attack remission under JAK inhibitor therapy further supports the rationale for JAK blockade in FMF.^7^

Similarly, Boyadzhieva et al. conducted a systematic review and found complete response in approximately 50% of FMF patients treated with JAK inhibitors, and partial response in the remainder.^7^ This suggests that earlier adoption of JAK inhibition in well-defined colchicine-resistant FMF may yield improved therapeutic outcomes.^13^

**Table 2** summarised all published Cases of FMF patients treated with JAK Inhibitors. All reported patients received tofacitinib (no published FMF cases yet for baricitinib, ruxolitinib, or upadacitinib) and were colchicine resistant. Most patients were refractory to biologics, such as IL-1 blockers. Improvement often occurs within months. Partial responders usually had resolution of symptoms, but persistent inflammatory markers, indicating subclinical inflammation.

**Table 2.** FMF patients treated with JAK inhibitors-published cases.

**Patient**	**Article link**	**Indication**	**Prior treatment**	**JAK inhibitor**	**Dose**	**Duration**	**Outcome**
1	Ref. ^13^	Refractory FMF	IL-1, TNF, IL-6 inhibitors	Tofacitinib	~10 mg/day	~4.5 mo	Complete
2	Ref. ^13^	Refractory FMF	IL-1, TNF, IL-6 inhibitors	Tofacitinib	~10 mg/day	~4.5 mo	Complete
3	Ref. ^13^	Refractory FMF	IL-1, TNF, IL-6 inhibitors	Tofacitinib	~10 mg/day	~4.5 mo	Partial
4	Ref. ^13^	Refractory FMF	IL-1, TNF, IL-6 inhibitors	Tofacitinib	~10 mg/day	~4.5 mo	Partial
5	Ref. ^22^	FMF	Not Reported	Tofacitinib	~10 mg/day	~4.5 mo	Complete
6	Ref. ^22^	FMF	Not Reported	Tofacitinib	~10 mg/day	~4.5 mo	Partial

Mechanistically, the impressive responses we observed may stem from the capacity of JAK inhibitors to intercept multiple cytokine signals simultaneously, thereby disrupting the downstream inflammatory cascade triggered by mutated pyrin. In recent studies, JAK1/TYK2 (which act downstream of the IFN receptor) were found to be essential for regulating IL-1β-mediated inflammation by network analyses and in silico tests supported by the fact that pyrin is interferon-inducible gene.^6,14^ While colchicine primarily modulates microtubule assembly and reduces inflammasome activation, it does not directly inhibit all cytokine signalling pathways.^15^ In contrast, JAK inhibition offers a broader suppression of inflammatory output by targeting shared intracellular signalling components.^16^ Kotyla reviewed the role of JAK inhibitors across autoimmune diseases and emphasised their capacity to block multiple proinflammatory cytokines at once.^17^ Moreover, Soriano et al. suggested that JAK inhibitors could be particularly valuable for monogenic autoinflammatory diseases that fail to respond to IL-1 or IL-6 blockade.^18^ Our clinical observations are consistent with this biological model: despite inadequate responses to colchicine and dapsone, the switch to a JAK inhibitor resulted in rapid and durable remission.

These results have several important clinical implications. First, they suggest that for patients with confirmed colchicine-resistant FMF and poor response to conventional therapy, transition to a JAK inhibitor may be a reasonable therapeutic option.^13^ Second, the oral route of administration and broad mechanism of action make JAK inhibitors a practical alternative to biologic injectables for patients preferring oral regimens. Third, while no adverse events occurred in our small cohort, clinicians should remain vigilant for known safety concerns, including increased infection risk, thrombosis, and potential cardiovascular events.^19,20^

Nevertheless, our study has limitations. The cohort size is small and lacks a comparator arm. The follow-up period, though sufficient to confirm sustained remission, does not allow assessment of long-term outcomes such as amyloidosis prevention. Without genotype– phenotype correlations beyond MEFV status or detailed biomarker data, generalisation of our findings must be approached cautiously. Future studies should include larger controlled cohorts, employ standardised disease activity indices (e.g., AIDAI, CRP, SAA),^21^ and investigate optimal dosing and safety monitoring protocols for JAK inhibitors in FMF.

These findings warrant validation in larger, long-term studies to define the efficacy, safety, and potential disease-modifying effects of JAK inhibitors in FMF.

## CONCLUSION

In conclusion, our experience with three colchicine-resistant FMF patients suggests that JAK inhibition may represent a meaningful therapeutic advancement, leading to sustained remission in cases refractory to conventional therapy. The rapid resolution of attacks and the absence of treatment-related adverse events suggest that JAK inhibition may represent a promising therapeutic option for patients with colchicine-resistant FMF, particularly in settings where biologics are either inaccessible or ineffective.

## Data Availability

The datasets used and/or analysed during the current study are available from the corresponding author on reasonable request.

## References

[1] Ozen S Kone-Paut I Gül A. Colchicine resistance and intolerance in familial mediterranean fever: Definition, causes, and alternative treatments. Semin Arthritis Rheum 2017;47(1):115–20. doi: 10.1016/j.semarthrit.2017.03.006. 28413100

[2] Ozen S Bilginer Y. A clinical guide to autoinflammatory diseases: familial Mediterranean fever and next-of-kin. Nat Rev Rheumatol 2014;10(3):135–47. doi: 10.1038/nrrheum.2013.174. 24247370

[3] El Hasbani G Jawad A Uthman I. Update on the management of colchicine resistant Familial Mediterranean Fever (FMF). Orphanet J Rare Dis 2019 Oct 15;14(1):224. doi: 10.1186/s13023-019-1201-7. 31615541 PMC6794843

[4] Salehzadeh F Enteshary A Moshkbar M. Colchicine plus Dapsone in Colchicine-Resistant FMF Patients. Case Rep Rheumatol.2019; 2019:2716127. doi: 10.1155/2019/2716127. 30729059 PMC6343160

[5] Hu X Li J Fu M Zhao X Wang W. The JAK/STAT signaling pathway: from bench to clinic. Signal Transduct Target Ther 2021;6(1):402. doi: 10.1038/s41392-021-00791-1. 34824210 PMC8617206

[6] Schwartz DM Kanno Y Villarino A Ward M Gadina M O’Shea JJ. JAK inhibition as a therapeutic strategy for immune and inflammatory diseases. Nat Rev Drug Discov 2017 Dec; 16(12):843-62. doi: 10.1038/nrd.2017.201. 29104284

[7] Boyadzhieva Z Ruffer N Burmester G Pankow A Krusche M. Effectiveness and safety of JAK inhibitors in autoinflammatory diseases: a systematic review. Front Med (Lausanne) 2022 Jun 27:9:930071. doi: 10.3389/fmed.2022.930071. 35833101 PMC9271622

[8] Jarneborn A Kopparapu PK Jin T. The Dual-Edged Sword: Risks and Benefits of JAK Inhibitors in Infections. Pathogens 2025;14(4). doi: 10.3390/pathogens14040324. PMC12030494 40333091

[9] Jamilloux Y El Jammal T Vuitton L Gerfaud-Valentin M Kerever S Sève P. JAK inhibitors for the treatment of autoimmune and inflammatory diseases. Autoimmun Rev 2019;18(11):102390. doi: 10.1016/j.autrev.2019.102390. 31520803

[10] Chaaban A Yassine H Hammoud R Kanaan R Karam L Ibrahim J-N. A narrative review on the role of cytokines in the pathogenesis and treatment of familial Mediterranean fever: an emphasis on pediatric cases. Front Pediatr 2024 Jul 26:12:1421353. doi: 10.3389/fped.2024.1421353. 39132307 PMC11310175

[11] Xue C Yao Q Gu X Shi Q Yuan X Chu Q Evolving cognition of the JAK-STAT signaling pathway: autoimmune disorders and cancer. Signal Transduct Target Ther 2023 May 19;8(1):204. doi: 10.1038/s41392-023-01468-7. 37208335 PMC10196327

[12] Banerjee S Biehl A Gadina M Hasni S Schwartz DM. JAK–STAT signaling as a target for inflammatory and autoimmune diseases: current and future prospects. Drugs 2017;77(5):521–46. doi: 10.1007/s40265-017-0701-9. 28255960 PMC7102286

[13] Karadeniz H Güler AA Atas N Satış H Salman RB Babaoglu H Tofacitinib for the treatment for colchicine-resistant familial Mediterranean fever: case-based review. Rheumatol Int 2020 Jan;40(1):169–73. doi: 10.1007/s00296-019-04490-7. 31813060

[14] Papin S Cuenin S Agostini L Martinon F Werner S Beer HD The SPRY domain of Pyrin, mutated in familial Mediterranean fever patients, interacts with inflammasome components and inhibits proIL-1β processing. Cell Death Differ 2007 Aug;14(8):1457-66. doi: 10.1038/sj.cdd.4402142. 17431422

[15] Dalbeth N Lauterio TJ Wolfe HR. Mechanism of action of colchicine in the treatment of gout. Clin Ther 2014 Oct 1;36(10):1465–79. doi: 10.1016/j.clinthera.2014.07.017. 25151572

[16] Schwartz DM Bonelli M Gadina M O’Shea JJ. Type I/II cytokines, JAKs, and new strategies for treating autoimmune diseases. Nat Rev Rheumatol 2016;12(1):25–36. 26633291 10.1038/nrrheum.2015.167 PMC4688091

[17] Kotyla PJ. Are Janus Kinase inhibitors superior over classic biologic agents in RA patients? Biomed Res Int 2018 May 10:2018:7492904. doi: 10.1155/2018/7492904. 29862290 PMC5971265

[18] Soriano A Soriano M Espinosa G Manna R Emmi G Cantarini L Current therapeutic options for the main monogenic autoinflammatory diseases and PFAPA syndrome: evidence-based approach and proposal of a practical guide. Front Immunol 2020 Jun 3:11:865. doi: 10.3389/fimmu.2020.00865. 32655539 PMC7325944

[19] Maqsood MH Weber BN Haberman RH Lo Sicco KI Bangalore S Garshick MS. Cardiovascular and Venous Thromboembolic Risk with Janus Kinase Inhibitors in Immune-Mediated Inflammatory Diseases: A Systematic Review and Meta-Analysis of Randomized Trials. ACR Open Rheumatol 2022 Oct;4(10):912–922. doi: 10.1002/acr2.11479. 35903881 PMC9555201

[20] Lamberg O Pandher K Troost JP Lim HW. Long-term adverse event risks of oral JAK inhibitors versus immunomodulators: a literature review. Arch Dermatol Res 2024 Dec 12;317(1):109. doi: 10.1007/s00403-024-03578-w. 39666160

[21] Veyssiere M Sadat Aghamiri S Hernandez Cervantes A; ImmunAID consortium; Henry T Soumelis V. A mathematical model of Familial Mediterranean Fever predicts mechanisms controlling inflammation. Clin Immunol.2023 Dec; 257:109839. doi: 10.1016/j.clim.2023.109839. 37952562

[22] Garcia-Robledo JE Aragón CC Nieto-Aristizabal I Posso-Osorio I Cañas CA Tobón GJ. Tofacitinib for familial Mediterranean fever: a new alternative therapy? Rheumatology (Oxford) 2019 Mar 1;58(3):553–4. doi: 10.1093/rheumatology/key384. PMID: 30535114 30535114

